# Low expression of aldehyde deyhdrogenase 1A1 (ALDH1A1) is a prognostic marker for poor survival in pancreatic cancer

**DOI:** 10.1186/1471-2407-11-275

**Published:** 2011-06-27

**Authors:** Christoph Kahlert, Frank Bergmann, Janine Beck, Thilo Welsch, Carolin Mogler, Esther Herpel, Shamik Dutta, Thomas Niemietz, Moritz Koch, Jürgen Weitz

**Affiliations:** 1 Department of General, Visceral and Transplantation Surgery, Im Neuenheimer Feld 110, 69120 Heidelberg, Germany; 2 Institute of Pathology, University of Heidelberg, Im Neuenheimer Feld 110, 69120 Heidelberg, Germany

**Keywords:** Pancreatic cancer, ALDH1A1, prognostic marker, proliferation rate

## Abstract

**Background:**

Aldehyde deyhdrogenase 1 (ALDH1) has been characterised as a cancer stem cell marker in different types of tumours. Additionally, it plays a pivotal role in gene regulation and endows tumour cells with augmented chemoresistance. Recently, ALDH1A1 has been described as a prognostic marker in a pancreatic cancer tissue microarray. The aim of this study was to reevaluate the expression of ALDH1A1 as a prognostic marker on whole-mount tissue sections.

**Methods:**

Real-time-quantitative-PCR (qRT-PCR) and Western blotting were used to evaluate the expression profile of ALDH1A1 in seven pancreatic cancer cell lines and one non-malignant pancreatic cell line. Immunostaining against ALDH1A1 and Ki-67 was performed on paraffin-embedded samples from 97 patients with pancreatic cancer. The immunohistochemical results were correlated to histopathological and clinical data.

**Results:**

qRT-PCR and Western blotting revealed a different expression pattern of ALDH1A1 in different malignant and non-malignant pancreatic cell lines. Immunohistochemical analysis demonstrated that ALDH1A1 was confined to the cellular cytoplasm and occurred in 72 cases (74%), whereas it was negative in 25 cases (26%). High expression of ALDH1A1 was significantly correlated to an increased proliferation rate (Spearman correlation, p = 0.01). Univariate and multivariate analyses showed that decreased expression of ALDH1A1 is an independent adverse prognostic factor for overall survival.

**Conclusions:**

Immunonhistochemical analysis on whole-mount tissue slides revealed that ALDH1A1 is more abundantly expressed in pancreatic cancer than initially reported by a tissue microarray analysis. Moreover, high expression of ALDH1A1 correlated significantly with the proliferation of tumour cells. Intriguingly, this study is the first which identifies low expression of ALDH1A1 as an independent adverse prognostic marker for overall survival in pancreatic cancer.

## Background

Although the incidence of pancreatic cancer amounts only to 3% of all tumours, it is a major cause of cancer-related death in Western countries [1]. Surgical resection remains the only potentially curative therapeutic option. At the time of initial diagnosis, only a minority of patients with pancreatic cancer are still in a curable resectable stage [2]. Even if a potentially curative resection can be performed, the five-year overall survival is low at 10-25% [2-4].

Current prognostic markers for curatively resected pancreatic cancer include lymph node status, tumour type and histological grade [2,4,5]. However, these prognostic markers only poorly predict metastatic progression or tumour response to medical treatment in the individual patient. Therefore new biomarkers are required in order to stratify patients into different risk categories, thus allowing a more specific treatment regimen.

Stem cell markers are a promising group of new biomarkers. In pancreatic cancer, several surface markers have been identified to provide a subpopulation of the tumour cells with so-called stem cell characteristics. These cancer stem cell markers include CD44 [6], CD24[6] and CD133 [7]. Their relevance as strong prognostic markers in pancreatic cancer has already been evaluated [8,9].

In this study, we have focused on aldehyde dehydrogenase 1A1 (ALDH1A1), which has been recently identified to label tumour stem cells in breast cancer [10], colon cancer [11] lung cancer [12] and head and neck squamous cancer [13]. ALDH1 belongs to the superfamily of NAD(P)(+)-dependent enzymes which metabolise a wide spectrum of endogenous and exogenous aliphatic and aromatic aldehydes [14]. It is distributed ubiquititously in many human tissues where it is localised in the cellular cytoplasm. By the formation of retinoic acid it acts as a pivotal modulator for gene regulation and cell differentiation [14]. Moreover, ALDH1 has a strong activity for detoxifying aldophosphamide, hence providing overexpressing cells with chemoresistance against cyclophosphamide [15].

Besides to gynaecological tumours and tumours of the respiratory tract [10,12,16], increased expression of ALDH1A1 in a pancreatic cancer tissue microarray has been recently described to correlate with a dismal prognosis [17]. On the contrary, increased expression of ALDH1 in ovarian cancer correlates with more favourable disease-free and overall survival [18]. Conversely, overexpression of ALDH1 in colorectal cancer is not related to differences in survival at all [19]. However, most of these results are based on tissue microarrays. This method is a very sophisticated tool to screen a large number of clinical specimens, but it might obscure essential findings by evaluating only small punched random samples from morphologically representative tissue areas. Hence, the aim of our study was to reevaluate the expression pattern of ALDH1A1 in pancreatic cancer on whole-mount tissue slides and to correlate these results with clinical and pathological data. Furthermore, ALDH1-positive non-pancreatic tumour cells have been found to have a high Ki-67 expression [12,20,21]. Therefore, we focused on the correlation between the expression of ALDH1A1 and the proliferation rate of tumour cells in pancreatic cancer samples. Our study demonstrates that high expression of ALDH1A1 is significantly correlated with the proliferation rate of pancreatic tumour cells. Intriguingly, this study is the first which identifies low expression of ALDH1A1 as an independent adverse prognostic marker for overall survival in pancreatic cancer.

## Methods

### Patients

Paraffin-embedded samples of primary pancreatic ductal adenocarcinoma from a consecutive series of 97 patients, who underwent tumour resection between 2002 and 2005 at the Department of General, Visceral, and Transplantation Surgery, University of Heidelberg, were included in this study. Previously, we had evaluated the prognostic significance of CD166 in this patient cohort [22]. No neoadjuvant radio- or chemotherapy was applied prior to surgical resection in any patient. After resection, 70 patients were subjected to adjuvant chemotherapy. The median observation period for overall survival was 18.3 months. Paraffin-embedded tumour samples were provided from the tissue bank of the National Centre for Tumour disease (NCT) Heidelberg. The tissue sampling and the analyses regarding potential prognostic markers were approved by the University of Heidelberg Ethics Committee. Table 1 displays the clinical and histopathological characteristics of the patients.

**Table 1:** Correlation between expression of ALDH1A1 in pancreatic cancer and clinical and pathological parameters

Characteristics	Number of cases	ALDH1A1 Low	ALDH1A1 High	P-Value
**Total **	97	59	38	

				

**Age**				

< mean 65 years	46	25	21	0.215

≥ mean 65 years	51	34	17	

				

**Gender**				

Female	44	30	14	0.18

Male	53	29	24	

				

**Lymph node status**				

N0	18	11	7	0.98

N1	79	48	31	

				

**Grading**				

1	4	2	2	0.83

2	57	34	23	

3	36	23	13	

				

**AJCC tumour stage**				

2	18	11	7	0.18

3	74	43	31	

4	5	5	0	

				

**R-Status**				

	88	53	35	0.70

	9	6	3	

ALDH1**A1**^Low^: percentage of positive cells ≤ median percentage of positive cells; ALDH1A1^High^: percentage of positive cells > median percentage of positive cells. P-Value evaluated by the Chi-square (χ^2^) test.

### Immunohistochemistry

Immunohistochemical staining for ALDH1A1 was performed as previously described [22]. Briefly, 1-2 μm sections of formalin-fixed, paraffin-embedded tumour samples were mounted on object slides (SUPERFROST^® ^PLUS microscope slides, Menzel, Braunschweig, Germany) and incubated at 37°C overnight. After de-waxing in xylene and ethanol, antigen retrieval was achieved by boiling in a microwave oven for 5 min (pH 6.0, 0.94 ml Antigen Unmasking Solution (Vector Laboratories, Burlingame, CA, USA)/100 ml distilled water) three times. After immersing slides in a 3.0% hydrogen peroxidase solution in methanol for 20 min in order to inhibit endogenous peroxidase activity, non-specific binding sites were blocked by pre-incubation with 10% normal goat serum (Vector Laboratories) in 1 mol/L PBS for 30 min at room temperature. Primary antibodies against ALDH1A1 (mouse IgG1, clone 44, BD Transduction Laboratories™, Europe, dilution 1:200, rabbit IgG, clone EP1933Y, Novus Biologicals, USA, 1:200, Rabbit polyclonal, Novus Biologicals, USA 1:50) and the IgG - negative control (mouse IgG, BD Pharmingen, Europe, 1:100) were incubated at 4°C overnight. Slides were loaded with secondary antibody coupled to peroxidase-conjugated polymers (EnVision^®^+ System, DakoCytomation A/S, Denmark) for 30 minutes. Afterwards, the immunoreaction was visualised by using AEC Substrate Chromogen (DakoCytomation A/S) according to the instructions of the manufacturer. Eventually, sections were counterstained with haematoxylin, dehydrated in graded concentrations of ethanol and mounted.

Staining against Ki-67 was performed at the tissue bank of the National Centre for Tumour Disease (NCT) Heidelberg by means of an automated immunostainer (Dako Autostainer A/S, Denmark) according to the manufacturer's instructions. Briefly, paraffin was removed with xylene and ethanol before the sections were de-masked for 15 min (pH 6.0, 0.94 ml Antigen Unmasking Solution, (Vector Laboratories)/100 ml distilled water). The slides were loaded with the primary antibody (Ki-67, Dako, A/S Denmark 1:200) and with the secondary antibody (Dako REAL Biotinylated Secondary Antibodies A/S Denmark) for 30 minutes, respectively. As the detection system, a streptavidin-peroxidase complex (Dako REAL Detection System) was used and the reactions were visualised by using Dako REAL AEC/H_2_O_2 _Substrate Solution (CHROM). Finally, the slides were counterstained with haematoxylin.

Evaluation of immunohistochemistry was performed by scanning the whole section at medium (50×) and high magnification (200×). Staining intensity and the percentage of positive tumour cells in the whole section were estimated by three independent researchers (CK, JB, NM) and two pathologists (CM, FB) on a blind basis. A multi-head microscope was used and consensus was reached for each slide. The staining intensity was classified as absent: 0, weak: 1, medium: 2 and strong: 3. The percentage of positive cells was categorised as 0: (< 5%), 1 (5-25%), 2 (> 25%-50%), 3 (> 50%-75%), and 4 (> 75%). By multiplying the value of the staining intensity with the categorised value of positive cells, a final immunohistochemical score was obtained. This scoring system has been suggested as a tool to evaluate prognostic biomarkers with broadly different levels of expression and intensity [23]. The proliferation rate of each tumour specimen was determined on a blind basis by categorising the estimated number of tumour cells stained positive against Ki-67 as 0: (< 5%), 1 (5-15%), 2 (> 15%-25%), 3 (> 25%).

### Cell lines

The following cell lines were used: SU8686, BxPC3, PANC-1, AsPC-1, MiaPaCa-2, ACBRI (all purchased from the American Type Culture Collection (ATCC), Manassas, VA 20108, USA), Colo357 (provided by T. Welsch) as well as T3M-4 (Cell Bank, RIKEN BioResource Centre, Japan). In June 2010, the cell lines were re-tested and re-authenticated by analysing cell samples for eight polymorphic short random repeat (STR) loci (Deutsche Sammlung von Mikroorganismen und Zellkulturen GmbH (DSMZ), Braunschweig, Germany).

Tumour cell lines were maintained in RPMI-1640 (Sigma, St. Louis, MO), supplemented with 10% (v/v) foetal calve serum (FCS), 100 U/ml penicillin and 100 μg/ml streptomycin. ACBRI cells were maintained in CSC Certified Complete Medium (CellSystems). All cells were cultured in a humidified atmosphere of 5% CO_2 _at 37°C.

### Total RNA isolation and real-time qPCR

Total RNA from the cell lines was extracted employing the RNeasy Mini Kit (Qiagen, Hilden, Germany) following the instruction manual. One microgram of total RNA was reverse transcribed using the miScript Reverse Transcription Kit (Qiagen). Ten nanograms of the resulting cDNA were used for quantification by qPCR (SYBR Green PCR Kit, Qiagen) in a Roche Light Cycler™ (Roche Diagnostics GmbH, Mannheim, Germany). Ready primer pairs specific for ALDH1A1 and 18S were purchased from Qiagen (Hs_ALDH1A1_1_SG QuantiTect Primer Assay, Cat. No. QT00013286 and Hs_RRN18S_1_SG QuantiTect Primer Assay, Cat. No. QT00199367). After an initial incubation at 95°C for 10 minutes, 40 cycles were carried out (15 sec at 95°C, 15 sec at 60°C, 30 sec at 72°C). All experiments were performed as triplicates. ALDH1A1 expression was normalised to 18 s and quantified as previously described [24].

### Western blotting

Cells were separated with 2 ml Accutase (PAA Cell Culture Company), washed with PBS and lysed in a cell lysis buffer (RIPA) with a protease inhibitor (Complete Mini, Roche Applied Science, Mannheim, Germany). Subsequently, they were disrupted by sonication and the protein concentration of the supernatant (centrifugation: 10 min, 11000 rpm, 4°C) was determined by a BCA kit (Thermo Scientific, Schwerte, Germany). Thirty micrograms of protein were separated on a 10% SDS-PAGE gel, transferred to a nitrocellulose membrane (BioRad, Munich, Germany) and incubated with antibodies against ALDH1A1 (Mouse IgG1, clone 44, BD Transduction Laboratories™) and α/ß-Tubulin (Cell Signaling Technology, Danvers, MA 01923, USA) with a dilution of 1:500. The horseradish peroxidase-conjugated secondary antibody anti-rabbit or anti-mouse (Sigma-Aldrich, Munich, Germany) was added at a dilution of 1:1000 after repeated washing with a wash/blocking buffer (PBS, Tween20 0.1% (v/v), skim milk powder 7,5% (w/v)). The antigen-antibody complexes were detected by using an ECL kit (Thermo scientific, Schwerte, Germany) according to the manufacturer's recommendations. All experiments were performed as triplicates.

### Statistical analysis

The software package SPSS, version 11.0 (SPSS, Chicago, IL, USA) was used for all calculations. The Chi-square (χ^2^) test was applied to examine the correlation between ALDH1A1 expression and clinical and pathological parameters. The Spearman correlation was employed for determining a correlation between the expression of ALDH1A1 and Ki-67. The Kaplan-Meier method was used to visualise overall and recurrence free survival curves, and differences between survival curves were assessed with the log-rank test for univariate analysis.

The Cox proportional hazards regression model was conducted on all covariates that showed a significant association with overall survival in the univariate analysis. The P-values of all statistical tests were two-sided and P < 0.05 was considered significant.

## Results

### Evaluation of specificity of ALDH1A1 immunohistochemistry

To validate the specificity of immunohistochemical staining against ALDH1A1, we repeated immunohistochemistry in 10 representative specimens with three different antibodies against ALDH1A1 and one IgG negative control. When comparing the immunohistochemical results of the three antibodies in sequential sections, they displayed similar cytoplasmic expression patterns in pancreatic cancer and in non-malignant cells, though the staining intensity of the polyclonal antibody was weaker than the staining intensity of the monoclonal antibodies (data not shown). These results, in addition to the Western blot analysis, confirmed the specificity of the anti-ALDH1A1 staining. For immunohistochemical evaluation of all clinical specimens, we used only the mouse monoclonal antibody (mouse IgG1, clone 44, BD Transduction Laboratories™, Europe), which had been previously used successfully [10,12,18,20].

### Expression of ALDH1A1 in pancreatic cell lines

**Figure 1:**
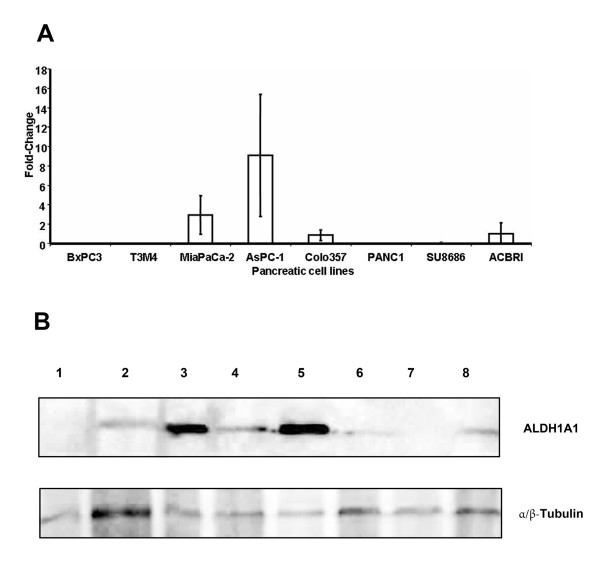
**(A) Gene expression analysis of ALDH1 by qPCR in seven pancreatic cancer cell lines and one non-malignant pancreatic cell line**. Fold-change (y-axis) of ALDH1 expression in pancreatic cancer cell lines compared to the non-malignant pancreatic epithelial cell ACBRI, respectively. (B) Western blotting analysis of ALDH1A1 in seven pancreatic cancer cell lines and one non-malignant pancreatic cell line (8). MW α/β-tubulin as loading control. 1: BxPC3, 2: T3M4, 3: MiaPaCa-2, 4: AsPC-1, 5: Colo357, 6: Panc1, 7: SU8686:, 8: ACBRI.

In seven pancreatic cancer cell lines and one primary pancreatic epithelial cell line, we assessed the expression of ALDH1A1 by real-time qPCR and Western blotting (Figure 1A and 1B). This analysis showed a varying abundance of ALDH1A1 in different pancreatic cell lines. By qPCR, ALDH1A1 was below the detection threshold in BxPC3, T3M4 and PANC1. In SU8686 and Colo-357, gene transcripts of ALDH1A1 were down-regulated by a 0.07- and 0.5-fold change respectively, compared to the non-malignant epithelial pancreatic cell line. In AsPC-1, ALDH1A1 was increased by 9-fold and in MiaPaCa-2 by 3-fold compared to ACBRI cells. These results were in good line with the Western blot analysis (Figure 1B). In summary, these data may be helpful for further *in vitro *studies when assessing the relevance of ALDH1A1 in pancreatic cancer.

### Expression of ALDH1A1 and Ki-67 in normal pancreatic tissue and pancreatic cancer cells

In non-malignant pancreatic tissue, adjacent to pancreatic cancer in 60 cases, we observed strong cytoplasmic expression of ALDH1A1 in the pancreatic islet cells, in the acinar epithelium and in the ductal epithelial cells. The staining intensity displayed a homogenous pattern: islet cells usually revealed a strong staining intensity, while acinar and ductal epithelial cells revealed a moderate staining intensity.

**Figure 2:**
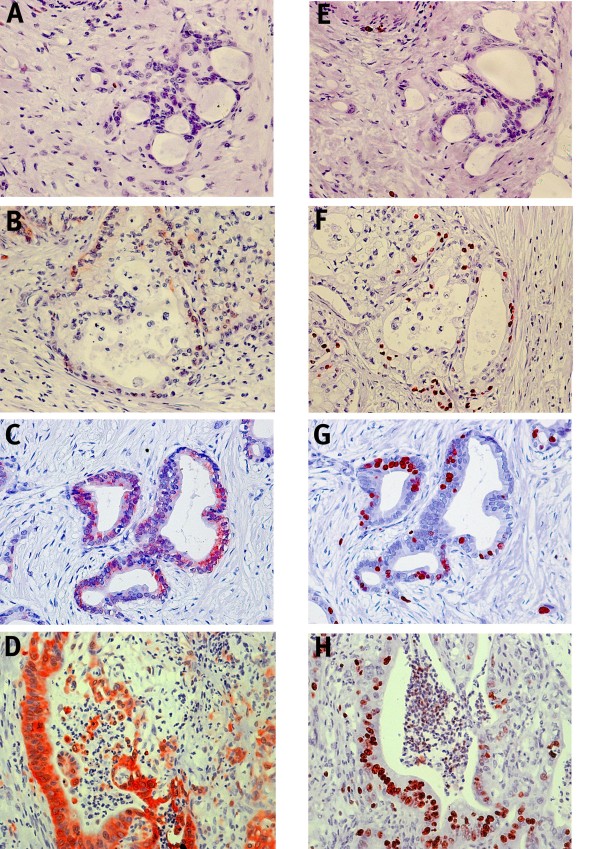
**Immunohistochemical analyses of ALDH1 in pancreatic cancer revealed a cytoplasmic expression varying from 0 - 3**. Comparing expression of ALDH1 and Ki-67 in corresponding sequential slides resulted in a positive correlation between strong expression of ALDH1 and a high proliferation rate. (A - D): pancreatic tumour samples displaying cytoplasmic expression of 0 (A), 1 (B), 2 (C) and 3 (D). (E - H): corresponding sequential slides with immunohistochemical staining against Ki-67. Original magnification 200×.

In pancreatic cancer, the expression of ALDH1A1 was confined to the cellular cytoplasm and occurred in 72 cases (74%), whereas it was negative in 25 cases (26%) (Figure 2A-D). There was no distinct difference in expression between the tumour center and the tumour invasion front. The distribution of staining intensity was as follows: intensity 0 in 25 samples (26%), intensity 1 in 13 samples (13%), intensity 2 in 29 samples (30%), and intensity 3 in 30 (30%) samples.

The percentage of positive cells was categorised as follows: 0 (< 5%): 25 samples (26%), 1 (5-25%): 18 samples (18%), 2 (26-50%): 16 samples (17%), 3 (51-75%): 18 samples (18%), 4 (> 75%): 20 samples (21%). As already previously reported in a small cohort of patient samples [25], high expression of ALDH1A1 was more often observed in well-differentiated tumours, though the correlation between expression and tumour grades did not reach statistical significance (Table 1).

The distribution of ALDH1A1 expression according to the immunohistochemical score was: 0: 25 samples, 1: 8 samples, 2: 10 samples, 3: 5 samples, 4: 11 samples, 6: 11 samples, 8: 3 samples, 9: 8 samples and 12: 17 samples.

The percentage of Ki-67 positive cells was categorised as follows 0 (< 5%): 27 samples (28%), 1 (5-15%): 27 samples (28%), 2 (> 15%-25%): 23 samples (23%), 3 (> 25%): 20 samples (21%). There was no difference in staining intensity among all samples.

### Relationship between the expression of ALDH1A1 and Ki-67

Recently, ALDH1 positive tumours have been found to have a high Ki-67 expression and to correlate positively with an increased proliferative capacity *in vitro *[12,20,21]. Therefore, we performed immunostaining against Ki-67 in consecutive sections and compared the percentage of Ki-67 positive cells with the immunohistochemical score of ALDH1A1 in pancreatic cancer (Figures 2E-H). The correlation between the proliferation rate and the immunohistochemical score of ALDH1A1 expression was shown to be significant (p = 0.01).

### Low expression of ALDH1A1 is an independent prognostic marker in pancreatic cancer

To compare the expression of ALDH1A1 with clinical and pathological parameters, samples were grouped as ALDH1A1^Low ^(immunohistochemical score ≤ median immunohistochemical score = 4) and as ALDH1A1^High ^(immunohistochemical score > median immunohistochemical score = 4). The Chi-square test revealed no significant correlation between ALDH1A1 expression to age, gender, lymph node status, grading, American Joint Cancer Committee (AJCC) tumour stage, or resection status (Table 1).

**Figure 3:**
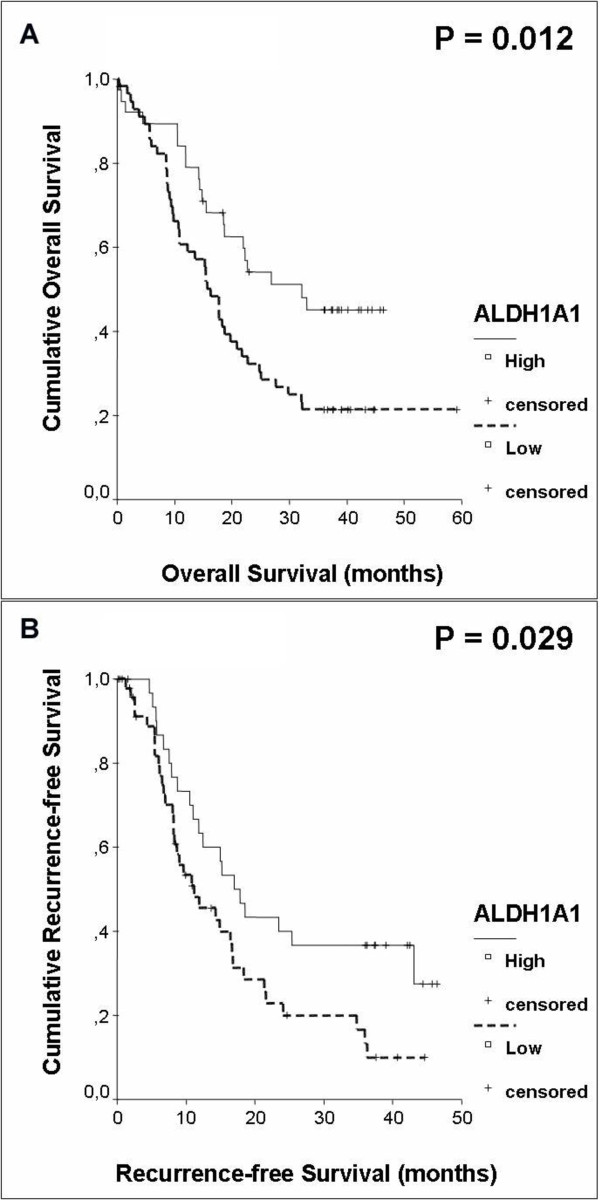
**Kaplan-Meier curves displaying (A) median overall survival and (B) median recurrence-free survival in 97 patients with pancreatic cancer and ALDH1^negative/low ^or ALDH1^high ^expression**.

By univariate analysis using the log-rank test, patients with ALDH1A1^Low ^tumours displayed significantly reduced median overall survival and recurrence-free survival compared to patients with ALDH1A1^High ^tumours (ALDH1A1^Low^: median overall survival 16.3 months, ALDH1A1^High^: median overall survival 32.2 months, p = 0.012, ALDH1A1^Low^: recurrence-free survival 11 months, ALDH1A1^High^: recurrence-free survival 17 months, p = 0.029) (Figure 3A and 3B). Furthermore, univariate analysis by the log-rank test demonstrated lymph node status, AJCC tumour stage and treatment with adjuvant chemotherapy to be significant prognostic parameters as described recently [22] (Table 2). Univariate analysis for the expression of Ki-67, performed by categorising the tumour samples into Ki-67^Low ^(proliferation rate ≤ median proliferation rate = 15%) and Ki-67^High ^(proliferation rate > median proliferation rate = 15%) showed no significant difference for overall survival and recurrence-free survival (Ki-67^Low^: median overall survival 21.8 months, Ki-67^High^: median overall survival 17.7 months, p = 0.27, Ki-67^Low^: recurrence-free survival 14 months, Ki-67^High^: recurrence-free survival 15 months, p = 0.78). These results are in accordance with previous observations [26,27].

**Table 2:** Univariate analysis for prognostic parameters in pancreatic cancer for median overall survival (OAS) and median recurrence-free (RFS).

Characteristics	RFS*			OAS		
	**No. of Cases**	**Time (months)**	**P-Value**	**No. Of cases**	**Time (months)**	**P-Value**

**Adjuvant Chemotherapy†**						

Not received	14	7	0.6	18	4.5	**0.0001**

Received	65	15		69	22.8	

						

**Lymph node status**						

N0	16	24	0.34	18	32.2	**0.04**

N1	64	12		78	17.7	

						

**AJCC tumour stage**						

2	16	24	0.28	18	32.2	**0.001**

3	59	12		73	18.7	

4	5	6		5	10.9	

						

**ALDH1A1**						

Low	47	11	**0.029**	58	16.3	**0.012**

High	33	17		38	32.3	

Total number of cases only 79, in 12 cases the exact time of tumour recurrence could not be specified.

† Type of adjuvant treatment in 9 cases is unknown.

Multivariate analysis (Cox regression model) of prognostic parameters for overall survival in pancreatic cancer

Multivariate analysis with the Cox proportional hazards regression model was performed to identify independent prognostic markers for overall survival. Low expression of ALDH1A1 was found to be an independent prognostic marker for overall survival (p = 0.004) in addition to AJCC tumour stage (p = 0.013) and treatment with adjuvant chemotherapy (p = 0.0001) (Table 3). Lymph node status was not included into the multivariate analysis due to its linear depending covariance with AJCC stage. Similar to the univariate analysis, ALDH1A1 was the only significant prognostic variable for recurrence-free survival in the multivariate analysis (p = 0.017). AJCC tumour stage and treatment with adjuvant chemotherapy had no significant impact as prognostic markers on recurrence-free survival by univariate analysis or by multivariate analysis.

**Table 3:** Multivariate analysis (Cox regression model) of prognostic parameters for overall survival in pancreatic cancer

Characteristics	Beta	Standard Error	Wald	Df	Relative Risk	95% CI of Relative Risk	P- Value
**ALDH1A1**							
Expression negative/low vs. High	-0.923	0.293	9.964	1	0.397	0.224-0.705	**0.002**

AJCC stage	0.891	0.288	9.592	1	2.438	1.387-4.286	**0.002**

Adjuvant chemotherapy vs. no adjuvant chemotherapy	-1.813	0.311	33.916	1	0.163	0.089-0.300	**0.0001**

### Subgroup analysis of ALDH1A1 expression in patients with adjuvant chemotherapy and no adjuvant treatment

**Figure 4:**
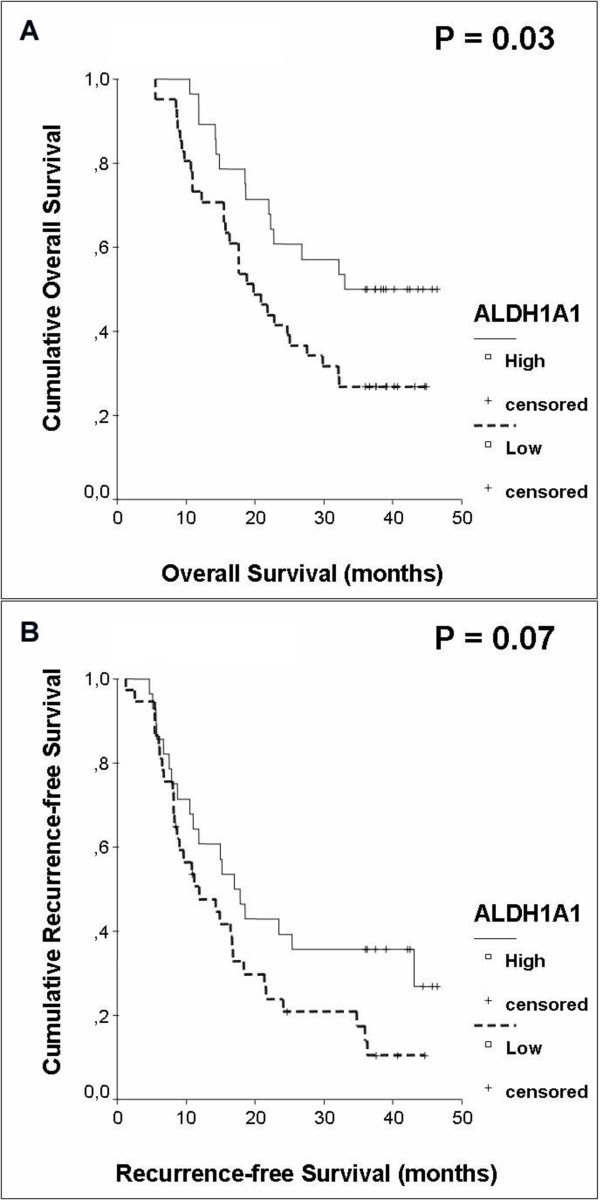
**Kaplan-Meier curves displaying (A) median overall survival and (B) median recurrence-free survival in a subgroup analysis of patients having received adjuvant chemotherapy**.

Adjuvant chemotherapy has improved survival in patients with pancreatic cancer significantly [28]. The identification of predictive biomarkers would be useful to stratify patients into different risk groups and to refine the selection of therapeutic agents being used in the postoperative setting. In this context, ALDH1 has been described as predictive marker in different types of tumour. In leukaemia and breast cancer, ALDH1 provides tumour cells with increased resistance against chemotherapeutical agents such as cyclophosphamide [15,29]. However, data also exist showing that increased expression of ALDH1 is associated with an improved response to chemotherapy in ovarian cancer [18]. For that reason, we performed a univariate analysis for the subgroup of patients having received adjuvant chemotherapy (n = 69) and for the subgroup of patients having not received adjuvant treatment (n = 19) to evaluate the role of ALDH1A1 as predictive marker for adjuvant chemotherapy. Nine patients with unknown adjuvant treatment were excluded. In this analysis, we identified strong expression of ALDH1A1 as a significant prognostic marker for more favourable overall survival in the subgroup of patients having been treated with adjuvant chemotherapy. Likewise, augmented expression of ALDH1A1 showed a trend for more favourable recurrence-free survival, though the level of significance failed (ALDH1A1^Low^: median overall survival 19.8 months, ALDH1A1^High^: median overall survival 33.0 months, p = 0.03, ALDH1A1^Low^: recurrence-free survival 11,9 months, ALDH1A1^High^: recurrence-free survival 17 months, p = 0.07) (Figure 4A and 4B).

A multivariate analysis including AJCC stage confirmed ALDH1A1^High ^in the subgroup of patients having received adjuvant chemotherapy as a significant prognostic marker (p = 0.0001) in addition to AJCC stage (p = 0.007). Univariate analysis with adjuvant chemotherapy was excluded for its linear depending covariance (Table 4).

**Table 4:** Multivariate analysis (Cox regression model) of prognostic parameters for overall survival in pancreatic cancer in the subgroup of patients having received adjuvant chemotherapy

Characteristics	Beta	Standard-Error	Wald	Relative Risk	95% CI Relative Risk	P-Value
**ALDH1A1**						
expression low vs. high	0.018	0.003	37.259	1.018	1.012-1.024	**0.0001**

AJCC stage	0.843	0.313	7.233	2.324	1.257-4.295	**0.007**

In the smaller subgroup of patients having not received chemotherapy postoperatively with adjuvant intentions (n = 19), different expression of ALDH1A1 had no significant impact as a prognostic marker on overall survival (p = 0.74) by univariate analysis nor on recurrence-free survival (p = 0.35) by univariate analysis.

## Discussion

In our study, we have demonstrated that low expression of ALDH1A1 is an independent prognostic marker for shortened disease-free and overall survival in ductal adenocarcinoma of the pancreas. These results are very conflicting to a recent published study by Rasheed et al., who have described increased expression of ALDH1A1 in pancreatic cancer to correlate with a dismal prognosis [17]. Since Rasheed et al. have evaluated the expression of ALDH1A1 by tissue microarrays, whereas we performed our immunohistochemical analysis on whole-mount tissue slides, methodological differences may explain these opposing results. However, by evaluating whole-mount tissue slides, we found ALDH1A1 to be expressed very heterogeneously within the tumour bulk. In fact, while Rasheed et al. claimed only 34% of the immunostained tumour samples as positive, we observed a much higher fraction of 74% of tumour specimen to be positive. Hence, using only 0.6 mm random tissue samples from morphologically representative tissue areas might obscure essential findings and result in an increased rate of false-negative results.

However, akin to the observations by Morimoto et al. in breast cancer [20] and by Jiang et al. in lung cancer cell lines [12], we have found a positive correlation between an increased expression of ALDH1A1 and a higher proliferation rate. Furthermore, we observed strong expression of ALDH1A1 more frequently in well-differentiated tumour samples. With these findings, it is tempting to hypothesise, as to why overexpression of ALDH1A1 correlates with a more favorable clinical outcome: assuming that conventional cytotoxic therapy aims mainly at the proliferating, differentiated cellular fraction, it can be conjectured that overexpression of ALDH1A1 is associated with a biological type of tumour which is more prone to chemotherapy. This hypothesis is supported by our findings, that increased expression of ALDH1A1 has been identified as a strong prognostic marker for improved clinical outcome in our subgroup analysis for patients having received adjuvant chemotherapy but not in the subgroup of patients having not received adjuvant treatment. Due to the low patient number in this subgroup of patients, however, this needs to be confirmed in further studies. In addition, our assumptions are in line with the clinical results of Chang et al., who reported a significant correlation between high expression of ALDH1 in ovarian cancer and response to chemotherapy [18]. Bearing in mind that ALDH1 increases chemoresistance against cyclophosphamide, one can argue that this chemotherapeutic agent is not part of the standard chemotherapy regimen against pancreatic cancer. Moreover, increased chemoresistance against cyclophosphamide by ALDH1 does not exclude increased chemosensitivity to other chemotherapeutic agents. Regarding our *in vitro *data, we have shown that MiaPaca2 cells have a strong expression of ALDH1A1. Intriguingly, Monti et al. have demonstrated that MiaPaca2 cells exhibit the strongest response to chemotherapy when exposed to combined treatment with gemcitabine and 5-FU [30]. These data would favour the hypothesis that increased expression of ALDH1A1 is not necessarily related to increased general chemoresistance but depends on the chemotherapeutical agent.

## Conclusion

In summary, our study describes low expression of ALDH1A1 as an independent prognostic marker for a poor clinical outcome in pancreatic cancer on whole-mount tissue slides. These data are conflicting with a previous report, which has claimed increased expression of ALDH1A1 to be an adverse prognostic marker in a retrospective study. Therefore, to evaluate the role of ALDH1A1 as a prognostic and predictive marker for tumour progression and response to chemotherapy in pancreatic cancer, standarised prospective studies with a larger number of patients are required.

## Disclosure of potential competing interests

The authors declare that they have no competing interests.

## Authors' contributions

CK designed the study, carried out immunohistochemistry, followed the patients and drafted the manuscript. FB evaluated the immunostaining and carried out immunohistochemistry. JB carried out immunohistochemistry, evaluated the immunostaining and followed patients. CM evaluated the immunostaining and analyzed the data. EH provided the clinical specimens for this study. TW raised and provided cell cultures. TN performed Western blotting and qPCR on cell cultures. SD performed the research and performed statistical analysis. MK participated in the design of the study. JW supervised research, analyzed the data and edited the paper. All authors read and approved the final manuscript.

## Pre-publication history

The pre-publication history for this paper can be accessed here:

http://www.biomedcentral.com/1471-2407/11/275/prepub
